# Mindful Climate Action: Health and Environmental Co-Benefits from Mindfulness-Based Behavioral Training

**DOI:** 10.3390/su8101040

**Published:** 2016-10-17

**Authors:** Bruce Barrett, Maggie Grabow, Cathy Middlecamp, Margaret Mooney, Mary M. Checovich, Alexander K. Converse, Bob Gillespie, Julia Yates

**Affiliations:** 1 Department of Family Medicine and Community Health, University of Wisconsin-Madison, Madison, WI 53715, USA; 2 Global Health Institute, University of Wisconsin-Madison, Madison, WI 53706, USA; 3 Nelson Institute for Environmental Studies, University of Wisconsin-Madison, Madison, WI 53706, US; 4 Cooperative Institute for Meteorological Satellite Studies, Space Science and Engineering Center, University of Wisconsin-Madison, Madison, WI 53706, USA; 5 Waisman Center, University of Wisconsin-Madison, Madison, WI 53705, USA; 6 UW Health Mindfulness Program, Integrative Medicine, University of Wisconsin, Madison, WI 53711, USA

**Keywords:** active transport, carbon footprint, climate change, co-benefits, environmental impact, health, meditation, mental health, mindfulness, sustainability

## Abstract

Greenhouse gases from human activities are causing climate change, creating risks for people around the globe. Behaviors involving transportation, diet, energy use, and purchasing drive greenhouse gas emissions, but are also related to health and well-being, providing opportunity for co-benefits. Replacing shorter automobile trips with walking or cycling, or eating plants rather than animals, for example, may increase personal health, while also reducing environmental impact. Mindfulness-based practices have been shown to enhance a variety of health outcomes, but have not been adapted towards environmental purposes. We designed the Mindful Climate Action (MCA) curriculum to help people improve their health while simultaneously lowering their carbon footprints. Combining mindfulness-based practices with the Stages of Change theory, the MCA program aims to: (1) improve personal health and well-being; (2) decrease energy use; (3) reduce automobile use; (4) increase active transport; (5) shift diet towards plant-based foods; and (6) reduce unnecessary purchasing. Mindfulness practices will foster attentional awareness, openness, and response flexibility, supporting positive behavior change. We plan to test MCA in a randomized controlled trial, with rigorous assessment of targeted outcomes. Our long-term goal is to refine and adapt the MCA program to a variety of audiences, in order to enhance public health and environmental sustainability.

## 1. Introduction

Several decades of scientific studies have confirmed that the planet is warming, the polar ice caps are melting, the sea level is rising, and the frequency and severity of weather events is increasing [1–5]. Climate change is due primarily to human activities [6–8]. Indeed the most recent Intergovernmental Panel on Climate Change (IPCC) report states “Human influence on the climate system is clear, and recent anthropogenic emissions of green-house gases are the highest in history”. Furthermore, emerging science suggests that climate change is leading to adverse health effects [9–15]. The stress of heat waves and droughts increases morbidity and mortality, as do poor air quality and food and water insecurity [16–19]. Floods threaten health by harming potable water and sanitation systems and by inundating homes, businesses, and agricultural land [20–23]. Climate change also accelerates the monumental and tragic loss of biodiversity [24–28], threatens food security, and increases the spread of infectious diseases such as malaria and gastrointestinal infections [29–32]. Because of rising sea levels [33–38], more than a billion people living on low-lying islands and coastlines will need to emigrate or adapt. Climate refugees are already undergoing resettlement in the United States [39]. Increasing waves of climate refugees also will put pressure on political, social and economic systems in neighboring countries [40]. Well-resourced nations will be stressed by increasing calls for aid and by numerous threats to the health of their own citizens [41–43].

While the magnitude and rapidity of climate change and the many effects on human societies cannot be predicted with precision, the expected scenarios are increasingly clear and quite dire. Strategies aimed at both mitigation and adaptation are urgently needed and should be undertaken on a scale commensurate with the threat. Rapid and massive transformation of energy systems toward carbon neutral technologies such as solar, wind, hydroelectric and geothermal is absolutely necessary but perhaps not sufficient. Even a greatly accelerated shift away from fossil fuels will leave us with a substantively warmer planet, with more unstable climate and weather patterns, and with increasingly serious threats to the health and happiness of virtually all peoples across the planet.

Our team acknowledges that macro-level efforts are urgently needed, but has concluded that these will need to be complemented by individual and community level efforts that can be tailored to specific needs and nuances of each locale. This paper focusses on using mindfulness-based trainings to modify individual-level choices and behaviors related to environmental sustainability, seeking to reduce carbon footprints while at the same time protecting and strengthening both mental and physical health, with potential for bringing lasting happiness.

Over many months, our multidisciplinary team designed Mindful Climate Action (MCA), an 8-week educational and behavioral training program. The MCA program pairs practices adapted from the empirically-supported mindfulness-based stress reduction (MBSR) program [44–47] with environmental and sustainability education. The program is aimed at fostering awareness of thoughts, feelings and sensations, which accordingly strengthens one’s connectedness to the environment and compassion for others.

The MCA framework is guided by stages of change theory [48–52] and motivational interviewing techniques [53–55], which have proven to be effective in modifying challenging behaviors such as drug and alcohol addiction. Each week, MCA participants will meet for 2.5 h in a class setting and will be asked to practice elsewhere 30 to 45 min daily. The vast majority of in-class time will be spent on mindfulness training practices, with approximately 30 min devoted to topics on climate change, energy use, and carbon and ecological footprints [56–60]. After the 8-week training sessions end, participants will be encouraged to continue their practice and will be contacted periodically, to obtain outcome data, to check in, and to encourage continued practice.

Mindful Climate Action is largely based on the promise of the health co-benefits that result from activities aimed at reducing greenhouse gases. For example, switching from coal-based power to electricity generated from wind, solar, hydro or geothermal could dramatically reduce respiratory and cardiovascular morbidity and mortality [61–66]. Shifting energy and transportation systems in a carbon neutral direction is necessary, but requires input from a variety of economic and political actors and will take time, as major infrastructure is involved. In contrast, the work that we propose is based on modifying individual behaviors that could have direct and immediate positive effects on both personal health and carbon footprint.

Active transport is an important example, which can have an effect on multiple levels, including personal, local, and global. By walking or bicycling instead of driving, an individual will reduce greenhouse gas emissions, reduce air pollution, and improve his or her health. From increased physical activity, the health benefits include personal fitness, positive mental health effects [67–69], and improvements in cardiovascular risk factors such as blood pressure, blood sugar and cholesterol levels, leading to lower mortality [70–72]. A study by co-author Maggie Grabow modelled outcomes from substituting short automobile trips with active transport and found substantive health benefits and economic savings from improving air quality and increasing physical activity [73]. Personal and societal health co-benefits could also occur from shifting individual dietary patterns away from meat and dairy and towards healthier and less environmentally impactful fruits, vegetables, and grains [74–76]. Finally, reductions in cycles of unnecessary purchasing may provide immediate economic benefits to individuals and lead to lower environmental impact for society.

The main focus of the proposed research project is to investigate whether and to what extent that MCA trainings can: (A) influence personal behaviors around energy use, transportation, dietary choices, and unnecessary purchasing (combined into a primary outcome of “carbon footprint”); and (B) increase human health and well-being, as self-reported on validated questionnaire instruments (general mental and physical health; self-efficacy; happiness; perceived stress; depressive symptoms). The specific aims are to determine whether and to what extent the MCA training program, when compared to a randomized control group, will lead to a significant:

1. Increase in knowledge of climate change, carbon footprint and energy use;
2. Reduction in household consumption of gas, electricity, and water;
3. Reduction in automobile and air transport (miles driven and flown);
4. Increase in active transport and physical activity (miles/minutes bicycled and walked);
5. Reduction of high-carbon footprint (animal-based) foods in diet;
6. Reduction in purchasing and consumption of non-essential goods;
7. Increase in self-reported general mental and physical health, self-efficacy, and happiness, with decreases in depressive symptoms and perceived stress.

## 2. Mindfulness-Based Behavioral Change

**Figure 1:**
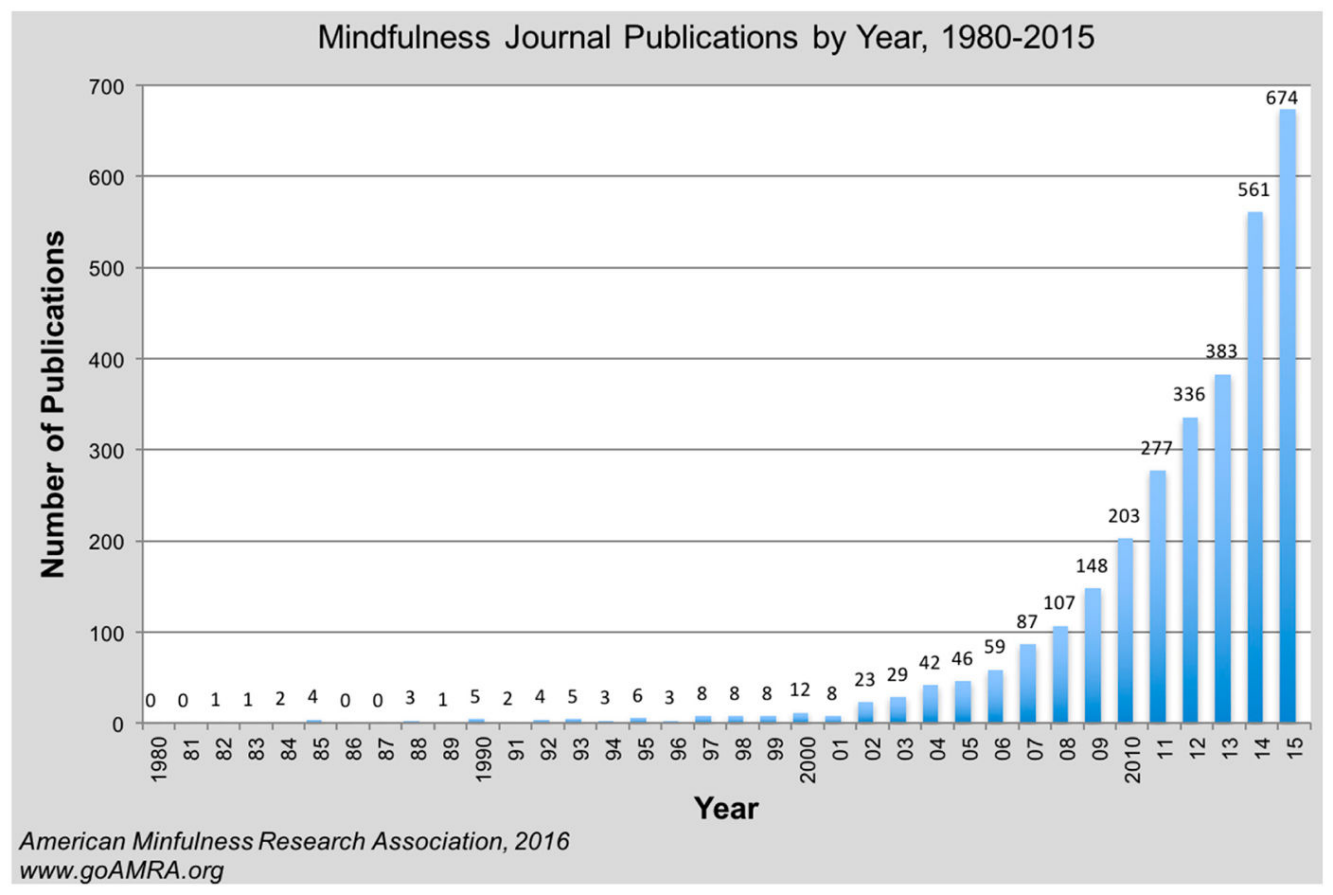
Number of Published Articles with “Mindfulness” in Title. ISI Web of Science publication search by David Black and the American Mindfulness Research Association. Used with permission.

Mindfulness was defined by Jon Kabat-Zinn as “the awareness that emerges through paying attention on purpose, in the present moment, and nonjudgmentally, to the unfolding of experience moment by moment” [47]. The last two decades have seen exponential growth of mindfulness research (Figure 1). Empirical research suggests that mindfulness-based interventions can reduce stress and anxiety, psychological distress, chronic pain, depression, and increase quality of life [77–82]. An NIH-sponsored randomized trial directed by Bruce Barrett found that MBSR trainings lead to lower incidence, duration, severity and impact of acute respiratory infections, and associated absenteeism [83–86]. A few observational studies report that higher levels of mindfulness may be associated with lower carbon footprints [87–91]. Amel and colleagues conduced a 100 person survey and found that: “acting with awareness was significantly positively correlated with self-reported sustainable behavior” [87]. Barbaro and Pickett conducted two studies, reporting that “mindfulness is significantly associated with pro-environmental behaviors” and that mindful traits of observing, nonreactivity, and connectedness “are particularly important” [88]. Jacob et al. reported results of a survey of 821 people, finding significant associations among mindfulness, ecologically sustainable behavior, and subjective well-being [90]. These observational studies support the association of mindfulness with sustainable behavior. The proposed MCA project will be the first experimental research to test the hypothesis that mindfulness-based environmental education can change specific behaviors related to carbon footprint.

### 2.1. Motivational Interviewing

Developed and tested within health care settings, “motivational interviewing” [53–55] methods linked to “stages of change” [48–52] theory are supported by many research studies and decades of clinical practice. Motivational interviewing (MI) is a specific type of counseling that seeks to strengthen and augment positive behavior change. Practitioners of motivational interviewing use open-ended questions, reflective listening, and genuine affirmation statements to allow clients to work through their own motivations, thoughts, feelings, and to develop, implement, refine and monitor their own plans for behavioral change. When clients use words that promote, affirm or explore positive change, the therapist listens and encourages, occasionally asking the client to explain, elaborate, or even move on to planning specific behavioral changes. When the client uses words to resist change or sustain unhealthy behaviors, the therapist gently redirects the conversation back to the client’s motivation for change. Thus, motivational interviewing focusses on listening, builds on positive change talk, and matches the therapeutic conversation with the client’s readiness for change. The MI framework is complementary with mindfulness training, and is beginning to be integrated into mindfulness programs; at the same time, MI therapists and counsellors are increasingly drawing from mindfulness theory and practice [92–97]. For the MCA program, instructors will incorporate nonjudgmental MI-style conversation as they assist individuals with behavior change.

### 2.2. Stages of Change Theory

Stages of change theory [48–52] asserts that people are most likely to undergo and sustain positive behavior change only when they are ready for it. According to this conceptual framework, the success of different types of interventions (counseling, teaching, rewards, positive and negative feedback) will largely depend on individual “readiness for change” and “where the person is at” with their own thinking, behaviors, and motivations. The generally accepted stages of behavioral change are:

1. **Pre-contemplation** Avoidance. Denying a problem, not considering change.
2. **Contemplation** Acknowledging that there is a problem, but struggling with ambivalence and inertia. Weighing pros and cons of sustained behavioral change.
3. **Preparation/Determination** Taking steps and getting ready to change.
4. **Action/Willpower** Making the change and living the new behaviors.
5. **Maintenance** Sustainably integrating behavior changes into one’s future life.

### 2.3. Four Climate Change Camps

**Figure 2:**
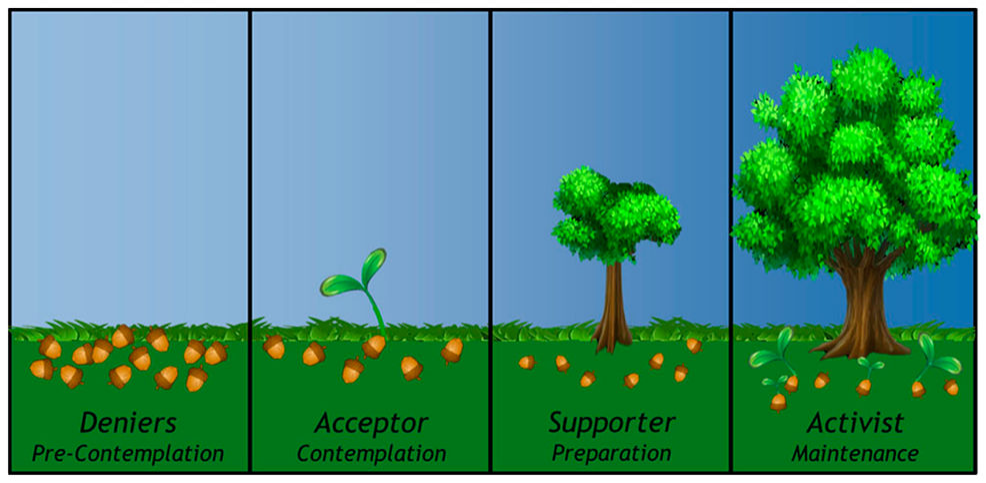
Four climate change camps (stages of change).

A simplified, but perhaps useful characterization of American society places people into four camps related to climate change: (1) Climate Change *Deniers* (pre-contemplation) are people who do not believe that climate change is a problem, and have no interest in learning about ways to mitigate or adapt; (2) Climate Change *Acceptors* (contemplation) understand that climate change is a problem, but are not at a place in their lives where they can commit to changing their own behaviors; (3) Climate Change *Supporters* (preparation/determination) who are interested in mitigating climate change, and may have begun to take positive steps, but are not yet committed; and (4) Climate Change *Activists* (maintenance) who have committed, and are substantively and sustainably reducing their own carbon footprints, and perhaps even more importantly, have begun to educate, motivate, and influence others. See Figure 2.

The MCA project will primarily target people in the two middle camps (Climate change *Acceptors* and *Supporters*), helping these people move from acceptance to positive action, and supporting positive behaviors among those already on their way towards sustained behavioral change. We expect that growing public awareness will gradually push Climate Change *Deniers* towards acceptance and then positive action, and that some people may become Climate Change *Activists*, teaching and influencing others to progress through the climate change camp stages towards sustained carbon footprint reduction and dynamic social change [98–103].

### 2.4. Multi-Level Behavioral Change

The proposed MCA project will incorporate these ideas and techniques in several ways at several levels. To begin with, we acknowledge that many people are in the pre-contemplative (Climate Change *Deniers* stage) with respect to climate change, and are not likely to join with us or benefit from MCA trainings. For those who are more ready for change (*Acceptors* and *Supporters*), a wide spectrum of knowledge, attitudes and behaviors (stages of change) and will be represented in the populations we hope to reach. In general, we expect that those who are able and willing to take part in this project will tend to be in the “contemplative”, “preparation”, or “action” stage of behavioral change in regards to anthropogenic climate change, and hence will be relatively motivated and reasonably capable of changing their energy-related behaviors to reduce their carbon footprints. Nevertheless, there will be substantive heterogeneity, and individuals will be met where they are—at their stage of change—with MCA trainings coming from a supportive rather than a directive standpoint.

### 2.5. Low Carbon Happiness

The proposed work aims to challenge the assumption that human health and happiness require large fossil fuel energy inputs. The underlying conceptual hypothesis is that people can feel healthy and rewarded without mindless consumption of high carbon goods and services. We recognize that traveling, over-eating, and unnecessary purchasing can bring short-term pleasure rewards. However, such rewards often are followed by periods of dissatisfaction or feeling “let down” that can lead to desire for more traveling, eating, or purchasing. Mindfulness training and practice will enhance ability to focus awareness on physical sensations, thoughts, emotions, and behaviors, which will in turn help to break the cycles of short-term pleasure reward. This will lead to new choices, behaviors and habits, and bring greater sustainable happiness. By incorporating techniques borrowed from motivational interviewing, we will invite people to explore their own motivations, and to rationally analyze and reinforce the broader lifestyle changes that they are making. All of this will help to support lifestyles that carry with them less environmental impact, leading to lower carbon footprint [87–90,104–112].

### 2.6. Co-Benefits

A large and growing literature regarding the health co-benefits that would naturally accrue from a variety of climate change mitigation efforts already exists [61–66]. Most has concentrated on society-level health outcomes that would be affected by changes to the agricultural, transportation and energy-producing sectors [113–118]. Several studies have concluded that substantive morbidity and mortality benefits would result from improved air quality, especially from the reduction of micro-particulates that would result from burning lower amounts of fossil fuels and firewood [63–66]. In the transportation sector, improvements in fuel-efficiency, increased use of public transport, and fewer diesel engines could all contribute to improved air quality and better health outcomes [115,118,119]. In the agricultural sector, soil conservation, carbon sequestration, reduced chemical and energy inputs, and a shift towards plant-based foods have all been proposed as ways to reduce the carbon footprint of our food supply chains [74,113,120,121]. While most of this work has been targeted at the co-benefits of industrial-scaled or societal-level changes, a small but growing literature is now looking at the effects of community or individual-level choices and behaviors [122–124]. For example, one study by MCA co-author Maggie Grabow found that substantive health and economic benefits could occur if people chose to walk or bike instead of drive for short local trips [73].

## 3. Mindful Climate Action Curriculum

### 

The MCA program will be piloted and tested in and around Madison, Wisconsin. Experienced instructors from the longstanding Mindfulness Program within the University of Wisconsin Hospital and Clinics’ Integrative Medicine program will teach the 8-week mindfulness sessions. The MCA sustainability curriculum will be incorporated into these sessions, employing a contemplative pedagogy fostering awareness and insight around connections between living sustainably, slowing climate change, and protecting local and global ecosystems. Participants will meet in class groups for 2½ h sessions, once per week for eight weeks. Approximately 2 h of each class session will be focused on mindfulness training, with awareness, compassion, and connectedness as central themes. Approximately 30 min of each class will engage participants in learning about the science of energy production and use, greenhouse gases, global warming, and climate change. Assigned daily homework practice will take between 30 and 45 min. In addition to the 8 weekly 2½ h classes, participants will be invited to a 6-h retreat to be scheduled on a weekend toward the end of the 8-week program. See Table 1.

Participants will receive about 26 h of contact time. Their daily home practice will be guided by audio recordings that lead them through their mindfulness practices including body scan, sitting meditations, gentle yoga, mindful walking, and mindful eating. Participants will be asked to practice for 5 or 6 days per week at home, choosing a practice time that works best for them (early morning, after work, or later in the evening). Classes normally involve a review of previously assigned homework, allowing for discussion of difficulties participants may have experienced. The class then progresses to new material, employing direct practice/experience with new techniques or skills. Experienced science instructors will lead the half-hour climate and energy sessions during the weekly class sessions. In general, each class will start with a mindfulness group practice lasting 30 to 45 min. This will be followed by 30 min of didactic environmental education, followed by 90 min or more of mindfulness practice.

The overarching learning objective for participants is to *make connections* across time and space by *reflecting on their daily, moment-to-moment choices and how they might impact the environment*. For example, how does eating rice, lettuce, or a hamburger connect to greenhouse gas emissions along the food supply chain? What are the consequences of wasting food? How does running a load of clothes in the dryer connect to an electrical utility power plant, clean air and clean water? How does the exhaust from our automobile’s tail pipe connect to rising CO_2_ levels in the atmosphere, to melting sea ice in the Arctic, and to rising storm tides in the tropics? And how do walking, bicycling, hanging clothes to dry, and eating lower on the food chain connect to better personal health? Meditation practices will be connected to these questions as they relate to daily choices and behaviors, both habitual and consciously directed. Specific formal mindfulness practices woven into the MCA curriculum include: Body scan; Breath awareness; Mindful eating; Mindful movement; Gentle hatha yoga; Sitting Meditation; Walking meditation; Emotion awareness; Thought awareness; Observing habitual behaviors; Observing reactivity; Conscious reflection; Reflection; Contemplation; Non-striving; Acceptance; Letting go; Compassion practice; Mountain meditation; Loving-Kindness meditation. In addition, informal practices that integrate mindfulness into activities of daily living will link participants’ awareness with the weekly themes of environment and sustainability.

#### Educational Resources

The MCA curriculum includes a variety of resources, including the locally-produced and accessible text *EnAct Steps to a Greener Living* [125], providing easy steps for people to “live a greener, more sustainable life”. The other two books that we plan to give to MCA participants are: (1) *How Bad Are Bananas? The Carbon Footprint of Everything* by MCA co-investigator Mike Berners-Lee [56], and *Climate Literacy: The Essential Principles of Climate* Science by the National Oceanic and Atmospheric Administration [3]. To begin to understand climate science, participants will watch a short YouTube video “*What is Climate?*” [126] from the National Academies of Science, the first of a 7-part video series. They will be encouraged to watch the rest of the series on their own. A variety of other printed materials, websites, videos and other resources will be made available to MCA participants. All educational resources will be accessible from the project web page, supplementing the 30-min instructional portion of each meeting.

#### Week 1: Mindful Eating; Healthy and Sustainable Diets

The first week of MCA class will engage participants in learning about the co-benefits of sustainable and healthy food choices. According to the United Nations Food & Agriculture Organization (UN FAO), “Sustainable diets are protective and respectful of biodiversity and ecosystems, culturally acceptable, accessible, economically fair and affordable; nutritionally adequate, safe and healthy; while optimizing natural and human resources” [127]. MCA participants will practice mindful eating, with focused attention on the sensations of tasting, chewing and swallowing, and with mindful contemplating about where their food is coming from, and what impacts food choices have on the ecosystem, and on personal health. We hypothesize that the enhanced awareness coming from these practices will lead to healthier and more sustainable eating habits, which we will assess using state-of-the-art methodology.

#### Week 2: Water Considerations for Sustainable Lifestyles

Water is integrally connected to personal health, food, energy use, and climate change. Participants in the MCA program will learn about water use, conservation and hydrological cycles, and will bring mindfulness to their daily use of—and interaction with—water. Participants will contemplate the many connections among water, energy use, food, climate, health and sustainability. According to the U.S. Environmental Protection Agency, “Approximately 4% of the nation’s electricity is used just for moving and treating drinking water and wastewater. Conversely, it takes 3000 to 6000 gallons of water annually to power just one 60-watt incandescent bulb for 12 h per day” [128]. Participants in the MCA training program will meditate on their own personal use of—and connection to—water as used for hydration, cleaning, sanitation, and food production.

#### Week 3: Walking Meditation, Exercise, and Active Transport

“Active transport” refers to physical activity undertaken as a means of *transport and not solely recreation*, such as walking or biking to work or the grocery store. Our goal is for participants to mindfully and incrementally incorporate active transport into their daily lifestyles by replacing shorter car trips with walking or bicycling. Through education and example, and by practicing mindful movement, participants will find the motivation to mindfully choose active transportation both for physical exercise and to get from one place to another. Walking meditation practice will be complemented by observation and reflection on how the body and mind feels when one walks or bikes to work or the post office, instead of driving. Participants will be invited to think about the bodily and societal consequences of walking and biking more, and driving less. From both ecological and personal health perspectives, substituting active transport for automobile transport is “low hanging fruit” with many co-benefits.

#### Week 4: Energy Conservation

Energy conservation is a triple win for participants: for their budgets, for their health, and for the planet on which they live. For example, by reducing their energy consumption, people will pay lower fuel bills, improve the air quality, and lower their carbon footprint. Participants in the MCA classes will learn about and reflect on these facts, and will be invited to bring mindfulness to their energy-related daily activities, such as using appliances and turning on and off light switches. A locally produce sustainability primer, *EnAct, Steps to Greener Living* [129], will provide participants with tips for conserving energy, being efficient, trying alternatives, considering renewable energy, and downsizing. The theme of reducing consumption will be reinforced in week #7. By learning non-judgmental acceptance of bodily sensations, MCA participants will accept a wider range of room temperatures, and will be able to reduce air conditioning and heating energy costs.

#### Week 5: Climate Connections across Time and Space

In keeping with the mindfulness principle of enhancing conscious awareness, the MCA course will encourage participants to think more deeply about how human activities support (or thwart) healthy ecosystems. Students will become familiar with carbon sequestration over the evolutionary time scale, and the relatively recent rise in the concentrations carbon dioxide, methane, and nitrous oxide. Atmospheric greenhouse gases such as these do not recognize national borders; climatic effects from fossil fuel combustion and agricultural practices in one country may be felt by citizens of other nations. This guiding principle of “connectedness” will be central to daily mindfulness practices, and will be reinforced by teachings from “*Climate Literacy: The Essential Principles of Climate Science*” [3] and “*How Bad Are Bananas? The Carbon Footprint of Everything*” [56].

#### Week 6: Ethical Considerations, Compassion, Vulnerable Populations, and Future Generations

A central finding of climate change science is that the populations least responsible for greenhouse gas emissions are the most likely to suffer harm [130–133]. Week 6 of the MCA curriculum will review the basic findings of climate change epidemiology [9,17,134–137] and introduce concepts of social justice. Instructors will foster compassion for vulnerable populations by asking participants to reflect on the broad scale of cause and consequence, compassion, and responsibility. Participants will be encouraged to think about the future and how today’s actions might affect future generations. Meditations will focus on how our consumption patterns affect vulnerable populations and future generations, and our possible responses, both as individuals and as a society. Participants will practice “loving kindness” meditations in class, focusing compassion first on themselves and their loved ones, and then on people they don’t know, or people that they don’t like, or that irritate them.

#### Weekend Retreat

The MCA course will include an all-day retreat (9 am to 4 pm) to be held on a Saturday or Sunday between week 6 and 7. This full day of mindfulness integrates all of the practices introduced six weeks, deepening the experience of participants. With most of the day spent in silence, MCA participants will practice mindful movement, sitting meditation, breathing exercises, awareness, and compassion. This immersion in an all-day retreat will foster contemplative insight and strengthen a sense of connectedness to other people, and to the world around us. We hypothesize that this will also serve to solidify attitudinal changes as well as choices, behaviors and practices relevant to ecological sustainability. For the last hour of the retreat, participants will converse about their experiences that day, and may discuss matters related to ecological sustainability. However, the retreat is primarily experiential, and will not include any specific instruction on energy use, climate change, or environmental impact.

#### Week 7: Personal and Planetary Well-Being; Purchasing and Consumption

The seventh weekly MCA class session will serve to integrate the themes of connectedness and compassion with insights gained during the retreat. Participants will be asked to think about and reflect on what, when, how, and why they choose to purchase various items. Concepts of desire and fulfillment, motivation, striving, purchasing, and the cycle of wanting and reward will be analyzed in terms of actual need, temporary pleasure, and lasting happiness. MCA participants will be asked to cultivate mindfulness before, during and after buying they purchase non-essential items, exploring how they feel before and after buying. The Week 7 class will include viewing and reflecting on “The story of stuff” video (http://storyofstuff.org/movies/story-of-stuff/).

#### Week 8: Time to Talk to Each Other; Conversation and Wrap-up

The final session of the 8 week MCA class will serve as a capstone for the MCA curriculum, allowing participants to explore how they intend to incorporate learnings and practices into their lives. Instructors will provide suggestions for how participants might continue mindfulness practice and ecological awareness in their everyday lives. Group discussion will focus on how the MCA class has affected participants, and how mindfulness practice and sustainable choices might help to sustain both personal and planetary health. Participants will be encouraged to reflect on their experience and share learnings and insights. Sitting and walking meditations, mindful movement and light exercise will be interspersed with group discussions. A closing guided meditation will focus on developing compassion for all living beings, and for our planet.

## 4. Research Plan

Our research plan is carefully matched to the Mindful Climate Action behavior change program. We will begin the research by pilot testing the MCA program, and will use qualitative data from interviews [138–140] and focus groups [141–143] to refine and streamline the content and delivery of education, training, and practice. Iterative qualitative testing and finalization of the MCA program will be followed by a randomized controlled trial (RCT), which will assess impact on targeted outcomes. The RCT experimental design [144] assures that all potential biases (influences from factors other than the trainings) will be balanced among the groups, allowing assessment of causal influence from the trainings. The mixed methods [145] design of iterative development and testing followed by RCT hypothesis-testing will incorporate best practice qualitative and quantitative research methods, with the goal of rigorously developing and investigating the effects of MCA trainings.

### 4.1. Populations Targeted

In the relatively near future, virtually all human beings will be affected by the effects of anthropogenic climate change. While everyone contributes to greenhouse gas emissions, the magnitude of the contribution (carbon footprint) varies widely across nations, peoples, and socioeconomic classes [57,74,108,109,146–148]. The people who are the least responsible for contributing to climate change are the most vulnerable to its effects [132]. In order to protect these marginalized populations, action must be taken to address those populations contributing a greater share of greenhouse gas emissions. In general, those in the higher socioeconomic classes have larger carbon footprints [149]. The MCA project will at first target middle to upper class American consumers in and around Madison, Wisconsin. These tend to be people with relatively high carbon footprints, but who also may value the environment, recognize the threat of climate change, and be motivated and capable of making choices and lifestyle changes to improve ecological behaviors in real and sustainable ways.

### 4.2. Sample Selection

For this initial project, we will recruit men and women aged 30 to 65 years, enrolling participants with assistance from our community partnerships. To be eligible, participants must:

- Own or co-own one or two automobiles,
- Own or co-own a house, duplex or condominium in the greater Madison, WI metropolitan area,
- Own and use a smart phone, and
- Be willing and able to carry out the many study activities, whether randomized to MCA trainings or the control group.

While we will not include or exclude people based on their knowledge or beliefs regarding anthropogenic climate change or their personal motivation to reduce carbon footprints, we expect that the very nature of this study will attract people who are open to climate change knowledge, mindfulness meditation, and carbon footprint reduction. In general, initial phases of MCA development, research, and dissemination will target those in the middle two “climate change camps” helping *Acceptors* transition to *Supporters*, and *Supporters* to become *Activists* (Figure 2).

### 4.3. Outcome Assessment

Table 2 depicts the outcomes and measurement tools to be employed. Comprehension of ecosystem, climate change, energy, and carbon footprint knowledge will be assessed by a college intro-level test (pre- and post-training). Electrical and gas use will be assessed directly by inspecting Madison Gas and Electric (MG&E) utility records. Water use data is available from the local water utility and will be acquired monthly. Miles driven by automobile will be assessed by self-report, and verified with odometer readings of primary vehicle(s) done by study staff. Dietary composition will be assessed by validated dietary intake food logs, with proportion of dietary calories from animal versus plant sources as the primary assessment. Carbon footprint of purchased nonessential goods will be assessed using a combination of a validated self-report tool and collection of receipts. Composite carbon footprint will be estimated using methods described below. We will also assess the health co-benefits of becoming more eco-minded, including several domains of mental and physical health, using several validated and well-established self-report measures. We will assess bodily exercise and active transport using *ActiGraph* accelerometers and the GPS-enabled smart phone *Moves* app [150], which tracks miles and minutes of walking, bicycling, and driving. To account for seasonal variations in energy use and to demonstrate sustained changes, participants will be followed for 12 months.

### 4.4. Carbon Footprint

As a shorthand for all greenhouse gases (e.g., CO_2_, CH_4_, N_2_O), the carbon footprint (CF) of “inputs” such as automobile driving, gas and electricity use, food intake, and purchasing of non-essential goods can be estimated with some degree of precision and reliability [57,120,123,146,148,159]. Individual-level carbon footprints (ICF) are assessed as ICF = ΣC_i_E_i_ where each term is the product of the degree of consumption (C) and the CO_2_ equivalent radiative forcing emission (E) of that input (i). Greenhouse gas emission (E) weightings applied to individual consumption levels (C) will be expressed in metric tons CO_2_-equivalent/year. Composite carbon footprints will be estimated using methods developed by Mike Berners-Lee and Small World Consulting [56,74,146,147], and compared to an online calculator developed at the University of California, Berkeley [152].

### 4.5. Innovation and Individual Orientation

The MCA program represents a potentially revolutionary approach to sustainability, as it aims to reduce societal demand for fossil fuels by changing the minds of individuals, one at a time. This proposed work will serve to highlight—and evaluate—the relationships of mindfulness, sustainability and personal health, and will test an innovative behavior change program using experimental methodology. It will provide quantitative data linking human health and well-being to objective behavioral outcomes important to environmental sustainability. The MCA program will employ mindfulness-based practices to foster behavioral and decision-making changes relevant to both sustainability and personal health. We hypothesize that this will lead to sustainable improvements in individual behaviors, which may in turn support social cohesion and environmental sustainability. To the best of our knowledge, this approach has not been tried before. The Mindful Climate Action program may represent a new ecological paradigm, as it begins with individual awareness, while aiming for health, sustainability, and low-carbon happiness. This may in turn strengthen feedback loops and ripple effects, influencing individual decisions and behaviors among friends, family, neighbors and coworkers. We hope that demonstrating the feasibility and testing the effectiveness of the MCA curriculum will be followed by a long-term growth trajectory, as our vision includes large-scale dissemination and implementation among individuals, households, community groups, faith-based groups, non-profits, educational institutions, small businesses, and larger corporations. After proof-of-concept and formal testing, we plan to establish a non-profit entity that will become sustainable through judicious marketing to educational institutions, health and medical care entities, and businesses interested in wellness, efficiency, cost-reduction, and ecological integrity. Nevertheless, the principles and practices incorporated in Mindful Climate Action are, like the air we breathe and the water that sustains us, shared collective resources that should be freely available to everyone.

## Figures and Tables

**Table 1:** Mindful Climate Action (MCA) weekly curriculum.

Week	MBSR Topic	Practices Learned	MCA Weekly Topic	Objective
1	Cultivating Beginner’s Mind and Non-judging *(Simple Awareness)*	Mindful eating Breath Awareness Body Scan	Mindful Eating: Healthy and Sustainable Diets	Develop awareness of the varying environmental and health impacts of food
2	Cultivating Non-striving *(Attention and The Brain)*	Loving Kindness Meditation	Water Considerations for Sustainable Lifestyles	Learn and understand the many implications of a changing climate on water scarcity
3	Cultivating Acceptance *(Dealing with Thoughts)*	Mindfulness within movement Hatha Yoga Walking Meditation	Walking Meditation, Exercise, and Active Transport	Gain understanding of multiple benefits of active transportation and motivate participants to choose active modes of transport whenever possible
4	Cultivating Patience *(Stress: Responding* vs. *Reacting)*	Sitting meditation	Energy Conservation	Make connections between daily activities and energy use and understand the benefits of reducing energy consumption; Learn how to relate to both pleasant and unpleasant experiences
5	Cultivating Letting Go *(Dealing with Difficult Emotions/Sensations)*	Sitting meditation	Climate Connections Across Time and Space	Develop awareness of the connectedness of all living beings and ecosystems
6	Cultivating Trust *(Mindfulness and Communication)*	Compassion for the conditioned mind Mindful movement Part 2	Ethical considerations and observed inequities in the causes and consequences of climate change	Develop awareness of broader impacts of climate change on those least responsible
(retreat)		Mindful movement Sitting meditation Breathing Exercises Fast Walking Laughing Meditation Compassion		Foster contemplative insight and strengthen a sense of connectedness to other people and the world around us
7	Cultivating Forgiveness *(Mindfulness and Compassion)*	Sitting meditation with choiceless awareness (Metta) Loving Kindness meditation	Personal and Planetary Well-Being; Purchasing and Consumption	Understand concepts of desire and fulfillment, motivation, striving, purchasing, and the cycle of wanting and reward in context of actual need, temporary pleasure, and lasting happiness
8	Sustaining Practice in Times of Transition *(Conclusion)*	Self-directed yoga and mindful movement	Mindful Climate Learnings	Discuss how to keep the momentum of mindful climate action going in every-day lives; Reflect on the significance of always making sustainable choices

**Table 2:** Mindful Climate Action outcomes that will be measured.

Outcome	Research Tool & Measurement
Knowledge of energy use, carbon footprint and climate change	College introductory level test (% correct at baseline and again after MCA trainings)
Carbon footprint from household utilities	Consumption of gas (therms), electricity (kilowatt-hours), and water (gallons) over a 12-month observation period
Carbon footprint from fossil-fuel transport	Automobile travel (verified by odometer readings over 12 months, and *Moves* app data); Travel by air, bus and train (miles travelled; verified by itineraries when possible)
Exercise and active transport	*ActiGraph* accelerometer (exercise) *Moves* app (active transport—miles, minutes biked & walked)
Dietary contributions to carbon footprint and health	Dietary Food Log (types and amounts of food consumed, assessed using University of Minnesota’s Nutritional Data System for Research software [151])
Carbon footprint estimate	CoolClimate Network Carbon Footprint Calculator [152] (an overall estimate of carbon footprint in tons of CO2/year) http://coolclimate.berkeley.edu/calculator
Consumer purchasing	Dollars spent on non-essential goods (assessed using a self-report tool based on validated consumer science instruments)
Health, wellness and happiness	SF-36—Highly validated, multi-purpose health survey [153] PSS-10—Perceived Stress Scale [154] PHI—Pemberton Happiness Index [155] CES-D—Center for Epidemiologic Studies Depression Scale [156] StPS—Stanford Presenteeism Scale [157] MSES—Mindfulness-based Self-Efficacy Scale [158]
